# Opportunities for personalised follow-up in breast cancer: the gap between daily practice and recurrence risk

**DOI:** 10.1007/s10549-024-07246-5

**Published:** 2024-02-26

**Authors:** Madelon M. Voets, Noa S. Hassink, Jeroen Veltman, Cornelis H. Slump, Hendrik Koffijberg, Sabine Siesling

**Affiliations:** 1 Department of Health Technology and Services Research, Technical Medical Centre, University of Twente, P.O. Box 217, 7500 AE Enschede, The Netherlands; 2 Department of Research and Development, Netherlands Comprehensive Cancer Organisation, P.O. Box 19079, 3501 DB Utrecht, The Netherlands; 3 Multi-Modality Medical Imaging, Technical Medical Centre, University of Twente, P.O. Box 217, 7500 AE Enschede, The Netherlands; 4 Department of Radiology, Ziekenhuisgroep Twente, Zilvermeeuw 1, 9609 PP Almelo, The Netherlands; 5 Department of Robotics and Mechatronics, Technical Medical Centre, University of Twente, P.O. Box 217, 7500 AE Enschede, The Netherlands

**Keywords:** Breast cancer, Follow-up, Real-world data, Risk of recurrence, Process mining, Resource utilisation

## Abstract

**Purpose:**

Follow-up guidelines barely diverge from a one-size-fits-all approach, even though the risk of recurrence differs per patient. However, the personalization of breast cancer care improves outcomes for patients. This study explores the variation in follow-up pathways in the Netherlands using real-world data to determine guideline adherence and the gap between daily practice and risk-based surveillance, to demonstrate the benefits of personalized risk-based surveillance compared with usual care.

**Methods:**

Patients with stage I–III invasive breast cancer who received surgical treatment in a general hospital between 2005 and 2020 were selected from the Netherlands Cancer Registry and included all imaging activities during follow-up from hospital-based electronic health records. Process analysis techniques were used to map patients and activities to investigate the real-world utilisation of resources and identify the opportunities for improvement. The INFLUENCE 2.0 nomogram was used for risk prediction of recurrence.

**Results:**

In the period between 2005 and 2020, 3478 patients were included with a mean follow-up of 4.9 years. In the first 12 months following treatment, patients visited the hospital between 1 and 5 times (mean 1.3, IQR 1–1) and received between 1 and 9 imaging activities (mean 1.7, IQR 1–2). Mammogram was the prevailing imaging modality, accounting for 70% of imaging activities. Patients with a low predicted risk of recurrence visited the hospital more often.

**Conclusions:**

Deviations from the guideline were not in line with the risk of recurrence and revealed a large gap, indicating that it is hard for clinicians to accurately estimate this risk and therefore objective risk predictions could bridge this gap.

## Introduction

Breast cancer is the dominant cancer diagnosis in women worldwide, comprising nearly a quarter of all cancers diagnosed. Additionally, breast cancer is the leading cause of cancer death in women [1]. Incidence numbers for breast cancer have been rising globally in higher-income countries over the past few decades, and in lower-income countries more recently [2, 3]. Factors explaining the rise involve the increased life expectancy and the associated change in reproductive patterns, including later age at first childbirth, decline in duration of breastfeeding and fewer full-term pregnancies. Furthermore, factors such as increased overweight, obesity and alcohol consumption are considerably increasing the risk of developing breast cancer [3]. Fortunately, as a result of earlier detection, improved treatment procedures and better access to healthcare, breast cancer mortality rates are steadily declining [2–4]. The personalisation of breast cancer diagnosis and treatment is a major factor in further bettering outcomes for patients whilst improving cost-effectiveness [5]. Additionally, to preserve quality of life and reduce costs, a gradual reduction of the intensity of therapy, based on individuals healthcare needs and risks, has been carried out successfully for breast cancer in the last decades. Despite the personalisation of treatment, follow-up after treatment is not yet individually tailored.

In the Netherlands, the average 5-year survival for invasive breast cancer has risen from 79% in 2000 to 88% in 2020 [6]. Consequently, more patients need of follow-up care after curative treatment. Early detection of locoregional recurrence (LRR) or second primary (SP) supported by imaging surveillance is one of the foremost aims of follow-up in addition to monitoring late treatment effects and psychosocial complaints (i.e. aftercare). The risk of recurrence is dependent on various patient, tumour and treatment characteristics, such as age at diagnosis, grade and size of primary tumour and type of surgery, and is known to change over time [7–9]. Of patients curatively treated for invasive breast cancer in the Netherlands, the risk of recurrence is highest in the second year post-diagnosis (3.9%), with on average 0.7% annual risk for local (LR) and 0.3% for regional recurrence (RR) [7]. Nevertheless, despite the de-escalation and personalisation of treatment, the Dutch guideline recommends annual mammography and physical examination in the first 5 years following curative treatment for most patients regardless of individual patient, tumour and treatment characteristics, unless in case of bilateral mastectomy [10]. However, several studies have previously evaluated guideline adherence and mainly reported a higher than recommended intensity of follow-up related to the application of radiotherapy, patient preferences, financial incentives and inadequate intervals, irrespective of patients’ individual risk of recurrence [11–13]. Additionally, nearly half of all recurrences are found by patients themselves between routine visits and are of similar severity as recurrences detected at routine visits [7, 14].

After 5 years, the further frequency of mammography is determined based on age and the presence of gene mutations, specifically BRCA 1 or 2. The Dutch guideline specifically mentions that the duration of follow-up should be decided in consultation between patient and physician, although it does not specify any basis for personalisation [10]. With the growing number of patients needing follow-up care, personalising follow-up schedules based on individual patient recurrence risk can help prevent patients from returning to the hospital for years with limited added value, lessen the burden on care resource capacity and healthcare budgets, whilst simultaneously reducing stress and other discomfort that patients may experience during follow-up [8, 15, 16].

In the past decades, the increasing pressure on healthcare budgets with increased the need to contain healthcare expenses, together with the replacement of payment for each individual activity by Diagnostic-Related Groups (DRG) and higher deductibles of health insurers might have all contributed to how surveillance in practice has changed [17, 18]. Moreover, it is unknown whether any changes over time in surveillance practice are related to the (perceived) risk of recurrent disease of the patient.

In 2015, Witteveen et al. developed the INFLUENCE 1.0 nomogram, estimating individual patient’s 5-year risks of LRR [19]. Since then, in 2021, the INFLUENCE 2.0 nomogram was developed to also include estimations of individual patient’s 5-year risks of second primary (SP) and distant metastasis (DM), plus incorporate additional relevant predictors [20]. Consequently, this nomogram can better support personalised follow-up strategies based on individual risk after curative treatment of non-metastatic breast cancer. Nonetheless, the nomogram is not yet integrated in the guideline or current clinical workflow and therefore information regarding patient’s individual risk of recurrence and consequently personalised surveillance pathways is limited.

Therefore, this study explores the variation in breast cancer follow-up pathways by using detailed patient-level data of a cohort of breast cancer patients over the past 20 years of a general hospital in the Netherlands to provide a complete overview of trends in surveillance pathways and guideline adherence. Additionally, we aim to assess the gap between daily clinical practice and risk-based follow-up using the INFLUENCE 2.0 nomogram.

## Methods

### Data sources

The retrospective cohort included patients over 18 years of age consecutively diagnosed with and treated for breast cancer of any type (International Classification for Disease-Oncology-10 C50) between 2005 and 2020 in a general hospital in the Netherlands. Patients were selected from the Netherlands Cancer Registry (NCR). The NCR is a population-based cancer registry hosted by the Netherlands Comprehensive Cancer Organisation (IKNL). Based on pathological notification through the national pathology archive (PALGA), data of patient and tumour characteristics, such as age, tumour histology, topography and stage, are registered by means of specially trained data managers. The cohort of patients from the NCR was subsequently linked to the electronic health records (EHR) to extract detailed hospital-based information on the imaging activities performed and delivered care procedures, i.e. detailed information regarding treatment and follow-up was collected.

### Patient and clinical activity selection

**Fig. 1:**
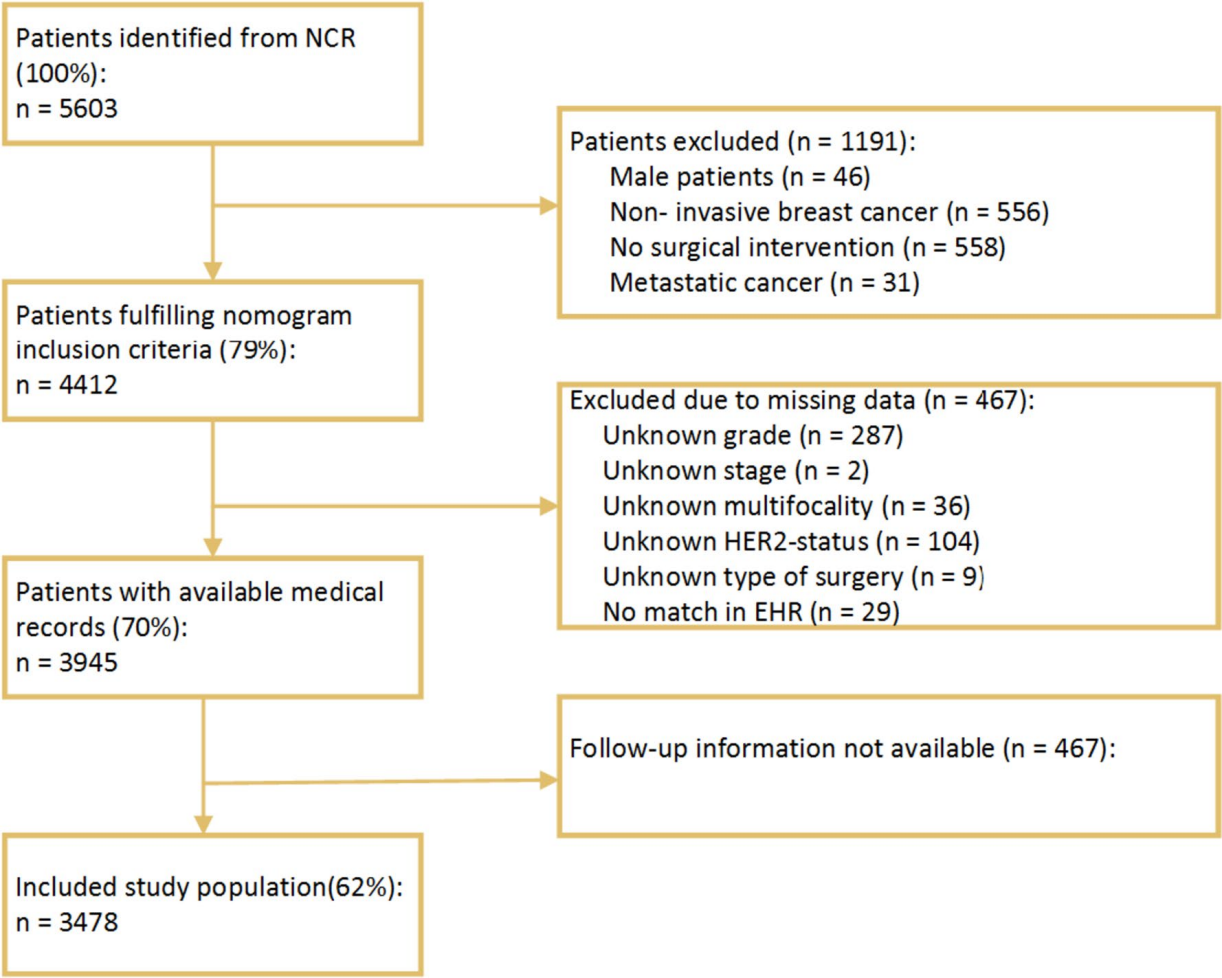
Patient inclusion based on the selection of the NCR and linkage to the EHR between 2005 and 2020

Patients with stage I–III invasive breast cancer (cM0) who had received surgical treatment with curative intent (no microscopic residue) were eligible for inclusion. Patients with unknown pathological TNM staging were classified according to their clinical TNM stage. The start of follow-up was defined as the first imaging activity following surgical treatment (i.e. mastectomy, or breast conserving surgery (BCS)). The end of follow-up was set at second or recurrent malignant diagnosis, death or last registered follow-up visit. Only the care activities delivered between the start and end of the defined period of follow-up were included in the analysis. One event log was created consisting of detailed information on the type and timing of the follow-up activity that was conducted for each individual patient. Follow-up activities included imaging studies such as mammography, ultrasound and magnetic resonance imaging (MRI). Patients selected from the NCR who could not be linked with the EHR were excluded (Fig. 1).

The individual overall 5-year risk of developing a recurrence was estimated by the INFLUENCE 2.0 nomogram. The nomogram estimated risk based on several patient, tumour and treatment characteristics, including age, type of surgery, grade, (nodal) stage, multifocality, hormone (oestrogen and progesterone) receptor status, HER2 status, radio-, chemo-, hormone- and targeted therapy. In case patients received more than one type of surgery, mastectomy was selected over BCS. To be included in the analysis of risk-based groups, patients had to have information on all of the input predictors of the INFLUENCE 2.0 nomogram, any missing predictors resulted in the exclusion of the patient (Fig. 1).

### Data analysis

For the analysis of the individual surveillance pathways, exploratory data analysis and process analysis techniques were employed. Process analysis is a technique to create a process map of which activities were performed, which patient was involved and what resources were used in an event log [21, 22]. This event log can be used to capitalise on the information in the mapping and explore the real-world utilisation of care processes and assess the underlying characteristics. Surveillance care activities were organised based on care activity identifiers and date. Additionally, the sequence and flow of imaging activities were visualised using Sankey diagrams. For data analysis, R 4.1.1 (https://www.r-project.org)* was used,* and for process analysis, R packages bupaR 0.5.2 and ggsankey were used [23–25].

For the analysis of the care activities for individual patients during their surveillance pathway, surveillance was divided into three intervals, because of the different nature of these intervals and thus expected variation between these intervals. The first interval was defined as the first 12 months after surgical treatment. This is the period in which patients can still receive treatment or monitoring before actual surveillance commences. The second interval is the second through fifth year after surgical treatment, in which the Dutch guideline suggest annual surveillance with mammography. The third interval is the period after the regular 5-year surveillance has ended and continuation of mammography is advised in some patient groups (annually below age 60 and between 60 and 75 biannually). A hospital visit was defined as any unique date an individual patient received one or more imaging activities, irrespective of whether a consultation and/or physical consultation occurred. Patients diagnosed after 1st January 2015 could not have completed 5 years of follow-up and were thus censored at some point.

## Results

### Patient population

Between 2005 and 2020, 5603 patients diagnosed with primary breast cancer were selected from the NCR. Only patients diagnosed from 2005 onwards could be included because the NCR started registering HER2 status after 2005. Of the 4412 patients who fulfilled the inclusion criteria, 467 had to be excluded due to missing data in one or more of the nomogram input predictors, primarily grade (287) and HER2 status (104). The remaining patients were linked to the EHR, 496 of which had incomplete or non-available follow-up information. Therefore, the included study population included 3478 individual patients. Figure 1 describes the process of patient inclusion.

Of the 3478 women included in the study, 1661 (48%) were younger than 60 years of age at the time of the primary diagnosis and the mean age was 60.1 (range 21–95) (Table 1). The majority of patients was diagnosed with a stage 1 tumour (61%) and no involved lymph nodes (64%). As for the surgical interventions, 1648 patients (47%) had received BCS, whilst 1830 patients (53%) underwent mastectomy. Most patients were treated with additional radiotherapy (62%) and no chemotherapy (66%). The mean follow-up time was 4.9 years, with a maximum of 15 years.

**Table 1:** Patient, tumour and treatment characteristics

Patient and tumour characteristics	*N*	%
Age at diagnosis^a^		
< 60	1661	48
60–69	990	28
70–79	598	17
≥ 80	229	7
Grade		
Low (1)	941	27
Intermediate (2)	1644	47
High (3)	893	26
Tumour stage/size		
pT1/ < 2 cm	2126	61
pT2/2–5 cm	1185	34
pT3/> 5 cm	167	5
Nodal stage/no. of positive lymph nodes		
pN0/Negative	2235	64
pN1/1–3	939	27
pN2/4–9	206	6
pN3/ ≥ 10	98	3
Multifocal		
Yes	589	17
No	2880	83
HER2 status		
Positive	487	14
Negative	2991	86
Hormone receptor status		
Positive	2937	84
Negative	541	16

Treatment characteristics	*N*	%
Type of surgery		
Breast-conserving	1648	47
Mastectomy	1830	53
Chemotherapy		
Yes	1187	34
No	2291	66
Radiotherapy		
Yes	2171	62
No	1307	38
Hormonal therapy		
Yes	1625	47
No	1853	53
Anti-HER2 therapy		
Yes	316	9
No	3162	91

^a^Mean age in years (± SD) = 60 (± 12); Median = 60; IQR: 51–69; Range 21–95

**Fig. 2:**
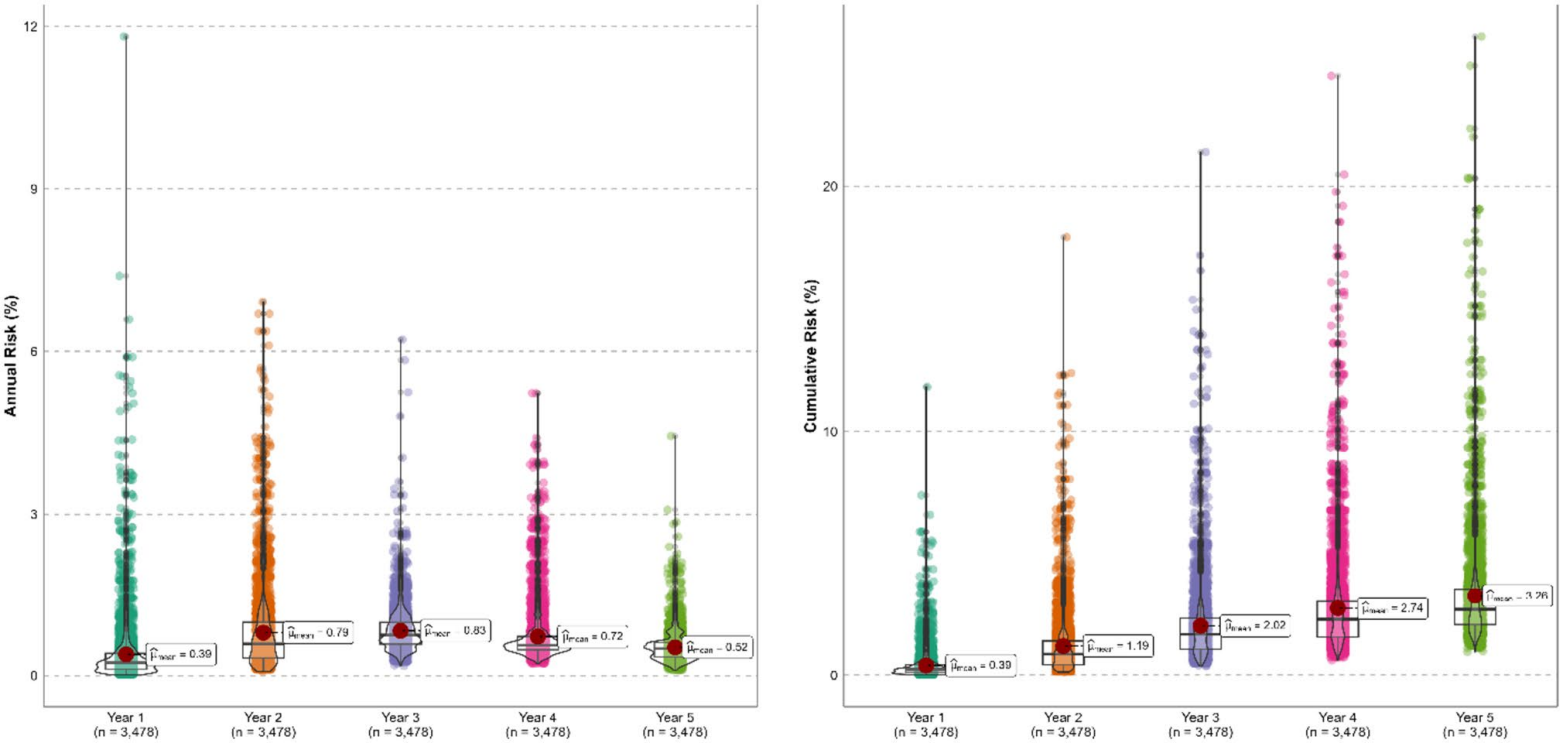
Annual (left) and cumulative (right) LRR risk predicted by the INFLUENCE 2.0 nomogram for all included patients

The predicted LRR risks for up to 5 years after the initial surgery using the INFLUENCE 2.0 nomogram varied based on the individual patient’s characteristics, but on average was lowest one year after finalising treatment (0.39%) and reached its highest value in year three (0.83%), before declining to a 0.52% risk in year five (Fig. 2). After 5 years, the cumulative risk of recurrence was on average 3.3%. The largest proportion (58%, *n* = 2015) had a risk below 3%, whilst 11% (*n* = 375) of patients had a cumulative risk larger than 5% (Fig. 2).

### Utilisation of imaging activities during surveillance visits

In the first 12 months following diagnosis, patients on average visited the hospital once and received two imaging activities (mammogram, ultrasound or MRI) during that visit. A quarter of all patients (*n* = 851, 25%) received one or more mammograms during their visit. In the following years, the intensity of surveillance steadily decreases. In the second year of surveillance, 89% of patients visited the hospital at least once, decreasing to 79%, 67% and 58% in the third, fourth and fifth years, respectively. In the course of 5 years follow-up, 25% of patients (*n* = 831) visited the hospital at least once every year, whilst 12% (*n* = 390) of patients received at least 1 mammogram every year. Considering the entire follow-up period, the average number of days between hospital visits was 345 (IQR 306–373).

**Fig. 3:**
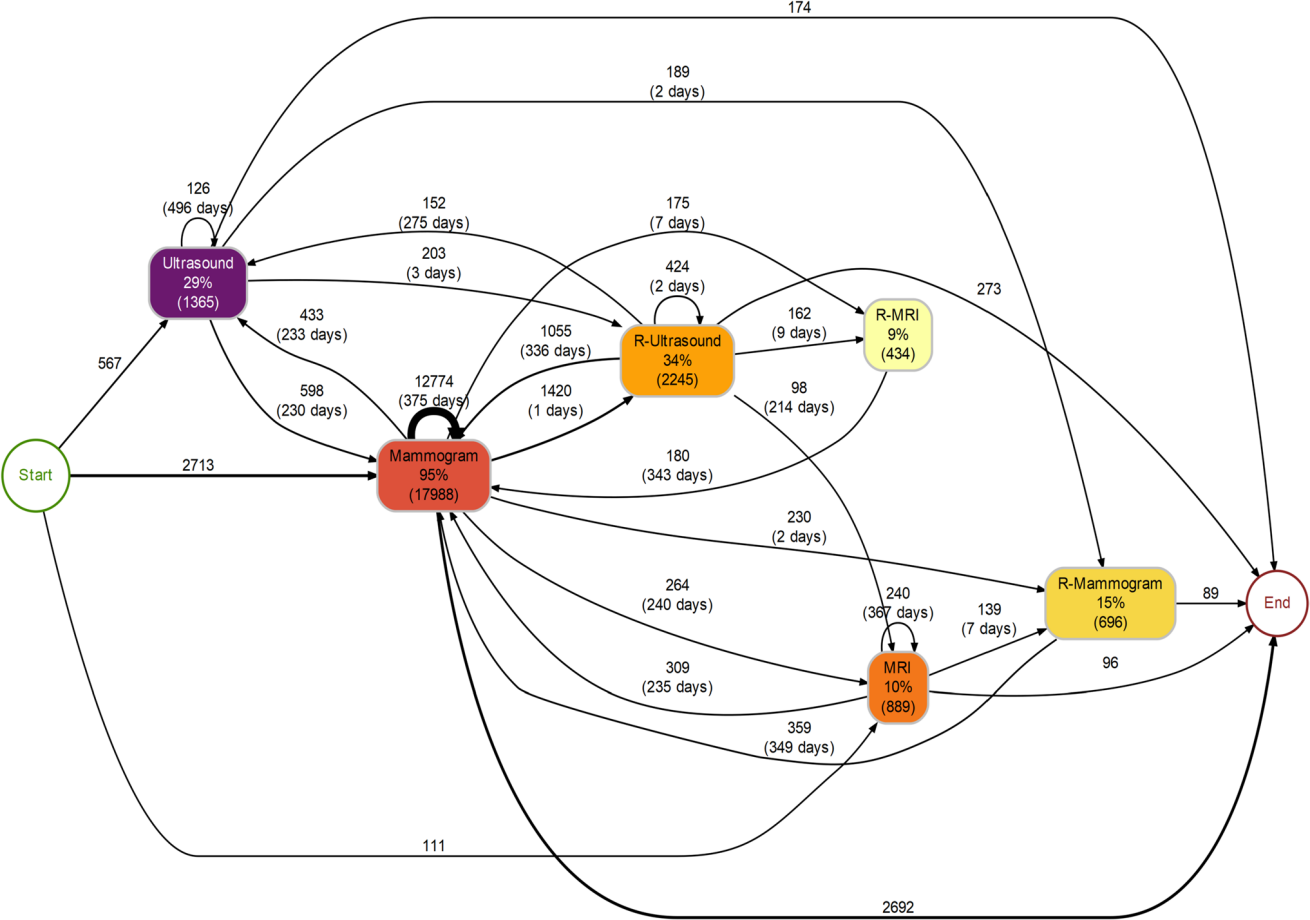
Sequential process map of follow-up. The numbers between brackets are the absolute number of activity instance executions, the percentages represent the proportion of patients in which the activity was executed. R-Mammogram, repeat mammogram within 40 days; R-Ultrasound, repeat ultrasound within 40 days; R-MRI, repeat MRI within 40 days

Furthermore, the mean 375 and 358 days between sequential mammograms and MRIs indicates these were performed annually (Fig. 3). Many imaging activities were performed between annual follow-up visits and the time between visits varies. The average number of days between consecutive ultrasounds was 496, whereas the average number of days between a mammogram and a sequential repeat ultrasound (*n* = 1420) was 1 day.

The total number of registered imaging activities was 26,389 during 21,234 hospital visits. Mammogram was the prevailing imaging modality, accounting for 70% of the imaging activities. Most patients begin their follow-up with a mammogram (*n* = 2713, 80%), frequently followed with another mammogram (*n* = 12,774, 79%) or ultrasound (*n* = 1365, 30%) (Fig. 3). MRI accounts for around 16% of all imaging activities and is the first imaging activity during follow-up in 3% (*n* = 111) of patients. Mammograms and ultrasounds which occurred within 40 days after the previous imaging activity are shown separately as ‘repeat’ (R) processes. Processes that comprise less than 5 per cent of connection between imaging activities were filtered out to enhance visual clarity.

**Fig. 4:**
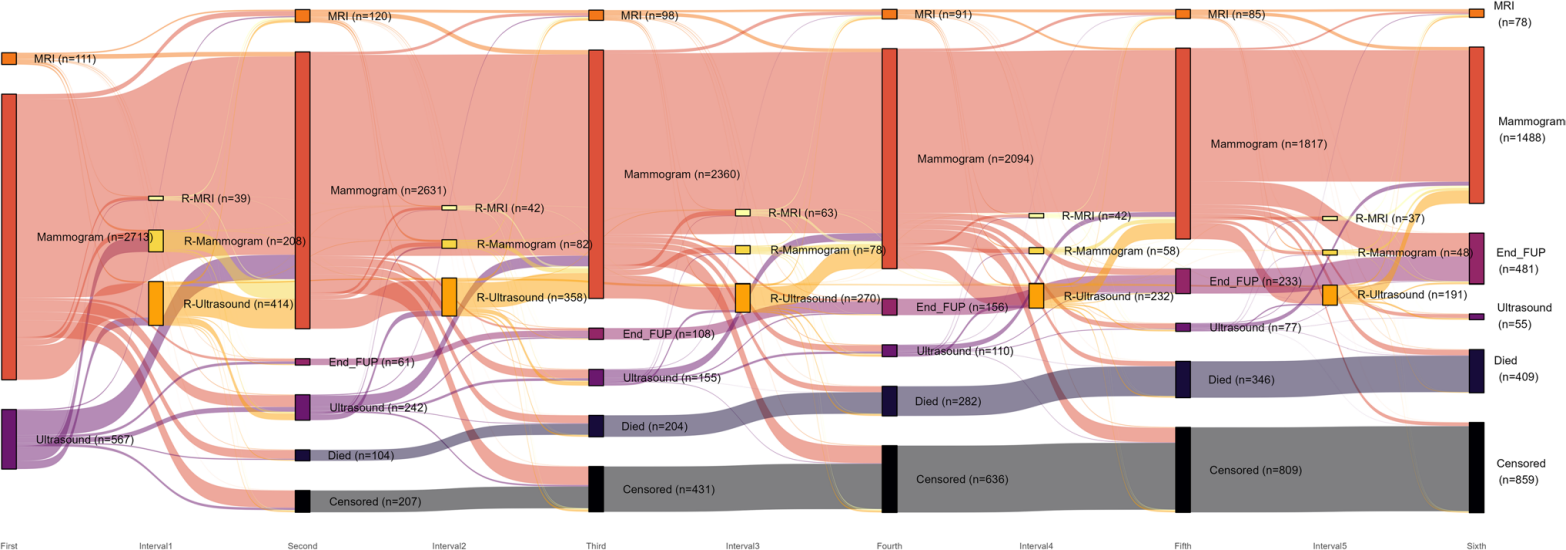
Sankey flow diagram describing the sequence of follow-up activities for all patients (*n* = 3478) during six years of follow-up and the repeat (interval) diagnostics between annual visits. *FUP* follow-up

To present a more detailed overview of the sequence of surveillance activities, the flow of patients between imaging activities during the first 6 years of follow-up is shown (Fig. 4). The width of the flows is proportional to the quantity of patients in that flow. Although the x-axis is related to the passing of time, it reflects the ordered sequence of activities, which are equally distanced across the axis. Mammography was the dominant imaging modality, followed by ultrasound and MRI. The group of patients not receiving follow-up grew as the years progressed. Within 40 days of the first imaging activity, 20% of patients were recalled to the hospital for a repeat diagnostic (mammogram, ultrasound or MRI). In the years thereafter, the group of patients recalled to the hospital between annual visits decreased to 14%. The number of patients who received an MRI at annual follow-up visits remained stable throughout follow-up, indicating that a group of patients was screened with MRI instead of mammography. By the sixth year, the number of mammogram was reduced by 46% and ultrasound by 91%, and the number of MRIs was reduced by 30% compared to the first year of surveillance.

## Discussion

Based on real-world extensive surveillance activity data, this study revealed that in a cohort of patients with stage I–III breast cancer, more surveillance activities occurred for patients with a predicted lower risk of recurrence compared to higher risk patients. Dependent on the individual, time-dependent and event-specific risk estimations from the INFLUENCE 2.0 nomogram, personalised follow-up schemes potentially reduce the number of clinical visits and associated costs. Our results illustrate the clinical variation and differences in surveillance pathways in the individual care pathways of patients. We observed a slight overutilisation (more than recommended) of breast cancer imaging during follow-up between 2005 and 2020, which was also observed in other studies [12, 26, 27]. Even though the patients in these studies were diagnosed in earlier years and from different hospitals in the Netherlands, the degree of overutilisation is less in this study than previously reported in terms of number of visits (3.9 compared to 1.1 visits in year 1 [26]) and follow-up time (mean 9.0 compared to 4.9 years [27]). Additionally, patients with more than average imaging activities were generally those with a lower risk of recurrence, estimated by the INFLUENCE 2.0 nomogram, as has previously been reported as well [13]. This indicated that without an objective risk estimation with a prediction model, it is difficult for healthcare professionals to assess risk and determine the optimal surveillance strategies for individual patients. Other studies corroborate these findings, stating that the number of observed surveillance visits differed significantly from the guideline [13] and reported the overuse of surveillance visits [26]. One study reported no significant relationship between the way of detection (i.e. routine visits or symptomatic discovery) of the LRR and the grade and stage of the LRR or the risk of DM, further underlining the de-escalation of follow-up [14].

In this cohort, the mean annual risk of recurrence is the highest in the second and third years post-diagnosis, which is consistent with previously reported studies [7, 8, 14, 28, 29]. Cause for the higher risk of recurrence early on in the follow-up period is the fact that more aggressive tumours recur earlier. According to Geurts et al., these types of tumours occur more often in younger (below 40 years), larger tumour size, higher grade, negative hormone status, BCS and > 3 positive lymph nodes [7]. Compared to their cohort, the cohort in this study has more low-grade tumours (60% grade I) and the majority of patients received mastectomy. Therefore, this cohort presumably includes fewer aggressive tumours, which explain why the highest mean annual risk of recurrence occurs in year three instead of the frequently reported second year post-diagnosis.

Moreover, this study has demonstrated that, in line with the guideline, mammography is the mainstay of follow-up care, as nearly every patient (96%) receives at least 1 mammogram during the surveillance period. Additionally, it was shown that a large group of patients receives an annual mammogram over the 5-year period (62%) and thus receives care according to the recommendations of the guideline. However, ultrasound and MRI were also frequently utilised. Yet, the average of 496 days between consecutive ultrasounds indicates that ultrasound may not be part of the annual follow-up strategies. In addition, the average of 1one day between a mammogram and a sequential repeat ultrasound suggests that ultrasound is preferred and performed quickly following an inconclusive mammogram. Although the exact indications and imaging results were not available in the data, one could imagine situations or patients where ultrasound or MRI may have been more suitable than mammography. For instance, for patients with an unfavourable genetic predisposition, screening with MRI is indicated before mammography, and in patients with mastectomy, ultrasound is more appropriate. Despite information regarding the genetic predisposition of patients not being available, patients who received MRI multiple times were generally younger (average 52 years, IQR 45–61). Similarly, the repeat diagnostics shown in Fig. 3 could well be explained by an inconclusive mammogram, after which an additional mammogram or different diagnostic such as ultrasound or MRI would be better suited.

Although the large sample size and level of detail of the data are valuable strengths of this study, the retrospective nature and the unstructured reporting of EHRs may have limited the correct interpretation of surveillance visits and imaging activities. The current completely free-text reporting makes identifying information regarding indication for an imaging activity, result interpretation and patient preferences consistently difficult. Complete and consistent reporting may be achieved through standardised structured reporting [30, 31] and not only eases the reuse of information [32, 33] and decreases of clinical workload [34], but is also directly linked to patient outcomes [35]. Therefore, the reasoning behind interval visits, i.e. whether these were initiated by the patient or the physician and whether the visit was routine or diagnostic was unclear, leading to a possible overestimation of the follow-up.

Furthermore, the INFLUENCE 2.0 nomogram has a defined set of predictors and is based on the NCR and therefore limited to the items collected in the NCR. Additional risk factors may influence the risk of recurrence, such as genetic predisposition or familial history [8]. Expanding the INFLUENCE 2.0 nomogram may increase model flexibility and clinical value. Additionally, other factors such as patient preferences, comorbidities and fear of recurrence could encourage deviating from a follow-up schedule based on the risk predictions by the INFLUENCE 2.0 nomogram [8]. Furthermore, future research into the potential factors contributing to the recurrence rate (e.g. BRCA 1/2, family history and breast density) separately for high-grade and low-grade tumours could be of great value.

Nevertheless, the results in this study outline the use of breast surveillance during follow-up based on real-world data, yet are not directly relatable to what would be a suitable personalised pattern of surveillance. This is mainly because the benefit of annual routine surveillance is still debatable given the many disadvantages regarding false positives, recall rates and increasing the psychological burden on patients, and individual risk predictions are not widely utilised in clinical practice. Despite the guideline recommendation for annual mammography and strong consensus between many different practice guidelines, this routine surveillance is not evidence-based but rather on the assumption that early detection of recurrence reduces breast cancer mortality [36, 37]. Furthermore, evidence of other surveillance intervals or the efficacy of different imaging modalities is lacking, whilst clinical practice is calling for personalised follow-up based on risk and patients’ needs [38]. Withal, this study demonstrates that follow-up is not as straightforward as the guideline describes, actual clinical practice exposes a considerable amount of variation and a large gap between daily practice and risk of recurrence. Personalised surveillance based on objective risk assessment using the INFLUENCE 2.0 nomogram therefore may provide an opportunity to support healthcare professionals in daily decision-making and may increase the efficiency of surveillance strategies.

## Conclusion

This article described the variation in surveillance for breast cancer patients by using patient-level data from the NCR linked with hospital-based EHR for a general hospital in the Netherlands between 2005 and 2020. Patients with a lower predicted risk of recurrence were associated with a higher number of hospital visits. Deviations from the guideline were not in line with the risk of recurrence and revealed a large gap indicating that it is hard for clinicians to accurately estimate this risk and therefore objective risk predictions could bridge this gap. This is unlikely to change unless objective validated risk predictions such as obtained through INFLUENCE 2.0 are widely implemented and used in clinical practice.

## Data Availability

The datasets generated during and analysed during the current study will be made available via the NCR upon request and after approval of a proposal from the date of publication. The plan for the statistical analysis will be made available by the corresponding author upon request.
